# Crocins with High Levels of Sugar Conjugation Contribute to the Yellow Colours of Early-Spring Flowering *Crocus* Tepals

**DOI:** 10.1371/journal.pone.0071946

**Published:** 2013-09-13

**Authors:** Angela Rubio Moraga, Oussama Ahrazem, José Luis Rambla, Antonio Granell, Lourdes Gómez Gómez

**Affiliations:** 1 Facultad de Farmacia, Universidad de Castilla-La Mancha, Campus Universitario s/n, Albacete, Spain; 2 Instituto Botánico, Universidad de Castilla-La Mancha, Campus Universitario s/n, Albacete, Spain; 3 Fundación Parque Científico y Tecnológico de Castilla-La Mancha, Paseo de la Renovación 1, Albacete, Spain; 4 Instituto de Biología Molecular y Celular de Plantas, Consejo Superior de Investigaciones Científicas-Universidad Politécnica de Valencia, Camino de Vera s/n, Valencia, Spain; Indian Institute of Science, India

## Abstract

*Crocus sativus* is the source of saffron spice, the processed stigma which accumulates glucosylated apocarotenoids known as crocins. Crocins are found in the stigmas of other *Crocuses*, determining the colourations observed from pale yellow to dark red. By contrast, tepals in *Crocus* species display a wider diversity of colours which range from purple, blue, yellow to white. In this study, we investigated whether the contribution of crocins to colour extends from stigmas to the tepals of yellow *Crocus* species. Tepals from seven species were analysed by UPLC-PDA and ESI-Q-TOF-MS/MS revealing for the first time the presence of highly glucosylated crocins in this tissue. β-carotene was found to be the precursor of these crocins and some of them were found to contain rhamnose, never before reported. When crocin profiles from tepals were compared with those from stigmas, clear differences were found, including the presence of new apocarotenoids in stigmas. Furthermore, each species showed a characteristic profile which was not correlated with the phylogenetic relationship among species. While gene expression analysis in tepals of genes involved in carotenoid metabolism showed that phytoene synthase was a key enzyme in apocarotenoid biosynthesis in tepals. Expression of a crocetin glucosyltransferase, previously identified in saffron, was detected in all the samples. The presence of crocins in tepals is compatible with the role of chromophores to attract pollinators. The identification of tepals as new sources of crocins is of special interest given their wide range of applications in medicine, cosmetics and colouring industries.

## Introduction

Carotenoids are a subgroup of isoprenoid compounds currently comprising over 700 structures. The oxidative cleavage of carotenoids leads to the release of a range of apocarotenoids that function as signalling molecules with diverse functions [1], [2], including the ubiquitous chromophore retinal, the phytohormone abscisic acid, and strigolactones, a group of C_15_ apocarotenoids attracting both symbiotic arbuscular mycorrhizal fungi and parasitic plants [3], [4] and exerting functions as novel plant hormones regulating shoot branching [5], [6]. Other apocarotenoids, probably involved in seed dispersal and pollinators attraction with high economic value are bixin in *Bixa orellana* [7] and crocetin (and their sugar conjugates known as crocins) in *Crocus sativus* (Fig. S1) [8], [9]. Crocetin and crocins are responsible for the colouring power of the saffron spice. Crocins are very soluble in water and exhibit a high colouring capacity. In addition, these compounds are powerful free radical quenchers and display a variety of health benefits which have been used in traditional medicine in various countries [10]. Interest in the therapeutical properties of these compounds has gained renewed attention due to their analgesic and sedative properties and effects to restrict growth of certain types of malignant cells, suggesting that saffron might be a potential anticancer agent [11], [12], [13]. In addition, clinical trials indicate that crocins are also beneficial in the treatment of depressions [14] and dementia [15]. However, commercial utilization of crocins from saffron is restricted by its limited availability which results in prohibitive prices.

Crocins accumulate at huge levels in the stigma of *C. sativus* and related species [16]. In *C. sativus*, crocetin is restricted to the stigma tissue [17]. The accumulation of this compound in the stigma begins early in development with levels rapidly increasing to reach a maximum in the red-immature stage to decrease thereafter with mature stigma and anthesis stages. The total content in crocins, the glucosylated crocetin derivatives, follows the same pattern as the levels of crocetin, but remains high in the mature stages up to anthesis [17]. Furthermore, these compounds have also been detected in the style and ovaries of senescent flowers and in the developing corm, where they are transported from the senescent stigma using the vascular system [18]. Crocins are formed by an unusual sequence of reactions which involves the cleavage of zeaxanthin and β-carotene [9], [17] followed by oxidative modifications and glucosylation reactions [19].

The flowers of *Crocus* (Iridaceae) show variability in tepal colour ranging from white, yellow, pale-brown, purple to lilac, mauve and blue. The *Crocus* genus comprises some 110–150 species with an old world distribution, primarily in Mediterranean-Europe and Western Asia [20]. The *Crocus* genus is divided into two subgenera, viz. subgenus *Crocus* comprising all species except one, viz. *C. banaticus* which is the sole member of the subgenus *Crociris*. The subgenus *Crocus* is further divided into two sections viz. section *Crocus* and section *Nudiscapus*, each again divided into different Series (Table S1). This subdivision of the genus is based on morphological as well as cytological characters [21]. In section *Nudiscaspus* the colour of the flowers ranges from yellow to purple and blue, plain in colour to variously suffused spotted striped patterns, which are characteristic for each species or subspecies. Within this section, series *Reticulati*, *Biflori*, *Orientales* and *Flavi* comprise autumn or spring flowering species, characterised by a three-partitate or multifid stigma. Yellow, white as well as blue flowers with a yellow throat are found in the spring-flowering species, while blue-lilac and white flowers are present in the autumn-flowering species. However, there is some variation, also within populations with regards to colour and especially markings [22]. Floral colours are due to biological pigments including a variety of different kinds of molecules, such as porphyrins, carotenoids, flavonoids and betalains. The colour variation caused by anthocyanins in the flowers of *Crocus* ranges from purple or brownish markings or stripes on the outer perianth segments to uniformly coloured lilac mauve, or blue flowers [23]. In addition to flavonols, carotenoids are most usually responsible for petal colour in the yellow-to-orange range. Several investigations of the carotenoid composition of petals have been reported [24]). Although the leaves of most plants have similar carotenoid compositions, the carotenoid composition in petals are species-specific, indicating different regulatory mechanisms affecting the carotenoid biosynthesis pathway in different plant organs [25]. Many flowers with white petals contain few carotenoid molecules, whereas the dark orange petals could accumulate up to several-fold the carotenoid content of leaves [26]. Genes controlling carotenoid biosynthesis as well as those involved in carotenoid degradation are known to be regulated at both the transcription and posttranscriptional level. Carotenoid can be catabolised to produce apocarotenoids by carotenoid cleavage dioxygenase (CCD) and 9-cis-epoxycarotenoid dioxygenase (NCED), which catalyze the cleavage of carotenoids at specific double bonds [1]. The CCD enzymes are grouped in CCD1, CCD4, CCD7 and CCD8 classes. Recent studies demonstrated that CCD4 contributes to white colour formation in chrysanthemum petals [27], [28], in peach fruits [29] and in potato tubers and flowers [30] by degrading coloured carotenoids into colourless compounds. In saffron stigmas however carotenoid catabolism seems to contribute to colour by increasing crocin levels and hence the colour in these tissues.

As crocins have been mainly studied in the stigmas of most *Crocus* species [16], we wished to determine whether a crocin biosynthesis pathway was also actively contributing to the yellow colour of spring *Crocus* tepals and how this pathway was regulated in this tissue.

## Materials and Methods

### Chemicals and Plant materials

Chemicals and reagents were obtained from Sigma-Aldrich unless otherwise stated. Tepals and stigmas at preanthesis and anthesis from *C. sativus* (Series *Crocus*), *C. chrysanthus* (Series *Biflori*), *C. ancyrensis*, *C. angustifolius* and *C. sieberi* (Series *Reticulati*), *C. olivieri* and *C. vitellinus* (Series *Flavi*) and *C. korolkowii* (Series *Orientales*) were collected from plants growing in the Botanical Garden of CLM (Albacete, Spain), immediately frozen in liquid nitrogen and stored at −80°C until required.

### Expression analysis

Total RNAs were isolated from internal tepals and stigma tissues by using Trizol (Invitrogen). First-strand cDNAs were synthesized by reverse transcription (RT) from 2 µg of total RNA using an 18-base pair oligo dT primer and a first-strand cDNA synthesis kit (Amersham Biosciences). The gene expression levels were evaluated using specific primer pairs for each gene. Primers for *PSY*, *CsPDS*, *CsCHY1*, *CstLcyBc*, *HMGR*, *OR*, *DXS*, *CsCCD4*, *CsCCD1a*, and *CsCCD1b* and amplification conditions have been previously described [16], [17], [31], [32]. Primers for *CsGLN2* were CsGLN2-F: 5′-TCGGTAGTTTGTCCAGTCTGA-3′ and CsGLN2-R: 5′-ACCGGTCGCAACGTGTGCGAG-3′. PCR was subsequently performed in 25 µl of a reaction mix containing 10 pmol of each gene-specific primer pair, 0.25 µl of first strand synthesis cDNA and 1 U of Taq DNA polymerase (Promega, Madison WI, USA). As an internal control, the mRNA level of the constitutively expressed ribosomal protein S18 (RPS18) was used [33], and genomic DNA from *C. sativus* was used as the positive PCR control (Note that the *HMGR*, *CsCHY1* and *DXS*, primers span an intron). A PTC-200 Programmable Thermal Cycler (controller) (MJ Research, Watertown, MA, USA) was used with an initial denaturation step of 94°C for 3 min, followed by 30 cycles of 94°C for 60 s, 60°C 20 s, 72°C for 40 s. The RT-PCR products were electrophoresed on a 1.5% agarose gel containing ethidium bromide and visualized under UV light. RT-PCR analyses were performed in duplicate using RNA from two different sets of plants. Results of the two replicates were similar, and therefore only one replicate is shown in the corresponding figures. For *C. sieberi* the yellow and purple parts of tepals were dissected and extracted separately.

### UV-Vis Determinations

An Agilent Cary® 60 Spectrophotometer UV-Vis G6860A (Agilent Technologies, CA) spectrophotometer, was used for spectra recording under the following conditions: start wavelength, 700 nm; end wavelength, 190 nm; data interval, 1 nm; scan speed, 460 nm/min.

### Extraction and analysis of carotenoids and apocarotenoids by HPLC-DAD

Apocarotenoids and carotenoids from stigma and tepals were extracted in a 2 ml microcentrifuge tube by grinding 1 stigma and 3 tepals from each species (fresh weight in Table S1) with a micropestle in 1 ml Tris-HCl (50 mM, pH 7.5) (containing 1 M NaCl), and incubated for 10 min on ice. One volume of chloroform was then added, mixed and the extract incubated on ice for an additional 10 min followed by centrifugation at 3,000 g for 5 min at 4°C. The lower chloroform phase was evaporated under N_2_ gas and the dried residues were stored together with the upper aqueous phases at −80°C until analysis of high-performance liquid chromatography (HPLC). All assays were performed in triplicate.

Two reverse phase HPLC methods were used for the analysis and detection of glycosylated compounds. Method A is a long method for the detection of glycosylated flavonoids and apocarotenoids, and was used as previously described [34]. A short method, method B, was used as a routine method for the detection of crocins and picrocrocin in saffron [17]. In both cases a Konik HPLC system (Barcelona, Spain) equipped with a Sugerlabor Intersil ODS-2.5-µm C18 column (250×4.6 mm) and connected on line to a photodiode array detector, with a dynamic range from UV-VIS region (200–700 nm) was used. The different carotenoid derivatives were identified on the basis of HPLC retention times and UV-visible light spectra [19], [35]. Method B was used to determine the area of the identified compounds in the different samples using three replicates. The results were presented in a table and as a dendrogram or heatmap; both have been implemented in MetaboAnalyst (http://www.metaboanalyst.ca/MetaboAnalyst/faces/Home.jsp).

Carotenoids were analysed by reverse phase HPLC as previously described [16] using a Konik HPLC system (Barcelona, Spain) equipped with a Sugerlabor Inertsil ODS-2.5-µm C18 column (250×4.6 mm) and connected on line to a photodiode array detector, with a dynamic range from ultraviolet to visible region (200–700 nm). Carotenoids were identified on the basis of HPLC retention times and UV-visible light spectra of authentic standards.

### UPLC-MS analysis

A UPLC/PDA/qTOF-MS instrument (micromass Q-TOF micro (Waters)), with the UPLC column connected on-line first to a PDA detector and then to the MS detector was used. Separation was performed on an ACQUITY BEH C18 column (150×2.1 mm i.d., 1.7 µm). The mobile phase consisted of formic acid∶ultrapure water (1∶1000, v/v; phase A) and formic acid∶acetonitrile (1∶1000, v/v; phase B). Gradient conditions were as follows: 80% A for 5 min, 80% to 25% A in 15 min, 25% to 0% A in 1 min, held at 100% B for 9 min, returned to 80% A in 1 min, and equilibrated for 4 min before the next injection. Flow rate was 0.4 mL min^−1^ and sample injection volume was 5 µL. Column and sample temperatures were 40°C and 10°C, respectively. UV-VIS spectra were acquired in the λ range 210–800 nm with a 1.2-nm resolution and 20 points s^−1^ sampling rate. MS analysis was performed by electrospray ionization in both positive and negative mode. Mass spectrometry conditions were as follows: capillary voltage, 3000 V; cone voltage, 30 V; desolvation temperature, 300°C; source temperature, 120°C; cone gas flow, 50 L h^−1^; desolvation gas flow, 500 L h^−1^. MS data were acquired in centroid mode in an m/z range of 100–2000 with a scan time of 0.32 s and an interscan time of 0.1 s. The MS was calibrated using sodium formate, and leu-enkephalin was utilized as the lock mass using a LockSpray exact mass ionization source. MassLynx version 4.1 and Q-tof Micro version 4.1 (Waters) were used to control the instruments and calculate the accurate masses.

### Extract hydrolysis and sugar analysis

Two hydrolysis methods were used for the analysis of the aglycones. In the first one, the glycosylated compounds from the aqueous extracts were hydrolyzed by adding acidified methanol (25 ml MeOH +1% (v/v) HCl) to the same volume of extracts and incubated at 90°C during 2 hr. In the second method an alkaline hydrolysis was performed as previously described [36].

Crocins peaks were purified in different batches using Method A. The samples were lyophilized, resuspended in 100 µl of water and incubated at 85°C with 1 M HCl for 120 min. After hydrolysis samples were cooled and neutralized with NaOH. Sugar analysis was performed after appropriate dilution of the neutralized samples, by converting first the neutral sugars to their corresponding alditol acetates [37] and later identified and quantified by GLC as described previously [38].

## Results

### β-carotene is the main carotenoid in the tepals of *C. ancyrensis* and is the precursor of the detected crocins

**Figure 1:**
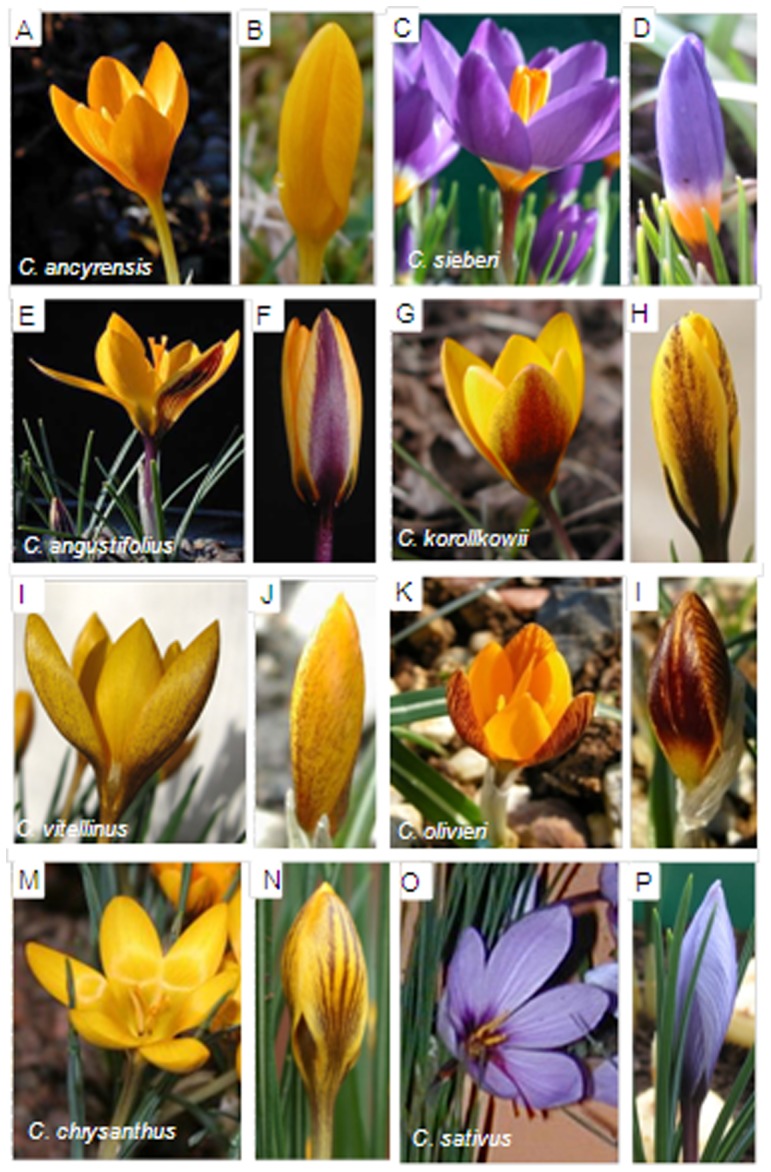
Open and closed flowers of *Crocus* species with yellow colours in tepals. Flowers of *Crocus ancyrensis* (A and B), *C sieberi* (C and D), *C. angustifolius* (E and F), *C. korollkowii* (G and H), *C. vitellinus* (I and J) and *C. olivieri* (K and L). Flower of *C. sativus* (M and N) used as reference.

**Figure 2:**
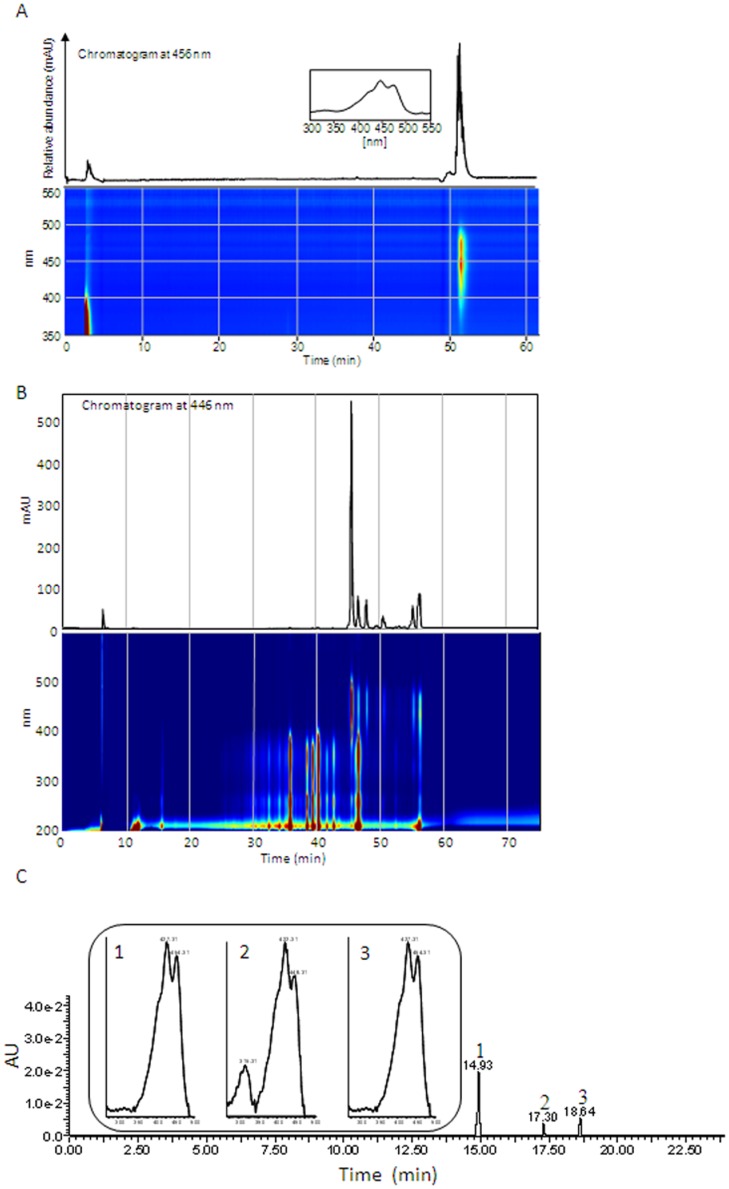
Carotenoids and apocarotenoids in the tepals of *C. ancyrensis*. A, HPLC chromatogram at 456*C. ancyrensis* tepals extracts. Compound eluting at retention time 52 min was identified as all-trans-β-carotene by comparison with an authentic standard. B, HPLC chromatogram at 446 nm of aqueous *C. ancyrensis* tepals extracts with method A. C, LC-PDA-ESI-QTOF-MS chromatogram corresponding to a hydrolyzed aqueous extracts from *C. ancyrensis* tepals recorded at 446 nm. The insets in C show the absorption profiles for the peaks detected after the hydrolysis reaction.

As a first step to determine the presence of carotenoid compounds as responsible for the yellow colour observed in the tepals of some *Crocus* species from the Series *Reticulati*, *Biflori*, *Orientales* and *Flavi* (Fig. 1A), the yellow tepals of *C. ancyrensis*, which is the only species showing a homogenous yellow colour without any other apparent colours in the external and internal tepals, were extracted as previously described [16] and carotenoids were analysed from the chloroform fraction. The HPLC analysis showed that β-carotene was the only carotenoid detected in the tepal extracts of *C. ancyrensis* under these conditions (Fig. 2A). Furthermore, the resulting yellow aqueous fraction was analysed for the presence of the saffron apocarotenoids crocetin, crocin and picrocrocin as well as for the presence of additional apocarotenoids. In addition to the presence of glycosylated flavonoids (detected at 250–350 nm), several other compounds were detected which exhibited spectra with absorption maxima between 418 and 446 nm resembling those of crocetin and crocins (C_20_) and of C_27_ apocarotenoids, all known to be yellow-orange [9], [39], [40], [41] (Fig. 2B). However, the analysis of the extracts at 250 nm did not reveal the presence of the saffron apocarotenoid picrocrocin, which was consistent with the absence of its precursor zeaxanthin in tepals.

In order to investigate the nature of these water soluble compounds, we performed acid hydrolysis of aqueous extracts followed by UPLC/PDA/qTOF-MS analysis (Fig. 2C). Three main compounds, not previously present in the aqueous phase were detected at 446 nm after the hydrolysis treatment: compound 1 (m/z 311.17 (crocetin-H_2_O); rt. 14.93; λ_max_ trans- 405, 427, 454 nm), compound 2 (m/z 329.10 (crocetin); rt. 17.30; λ_max_ cis- 405, 422, 446 nm) and compound 3 (m/z 311.17 (crcetin-H_2_O), rt. 18.64; λ_max_ trans- 405, 427, 450 nm) [18]. Similar results were also obtained after alkaline hydrolysis of the same extracts (Fig. S2A).

When zeaxanthin acts as the precursor of crocins, the compound 4-hydroxy-2,6,6-trimethyl-1-cyclohexene-1-carboxaldehyde (HTCC), is generated and T glucosylated to picrocrocin. HTCC was not detected in the HCl hydrolysed extract, rather three main UV-absorbing compounds with max absorption spectra at 276 nm (peak 1), 281 nm (peak 2) and 290 nm (peak 3) were present before and after the acid hydrolysis (Fig. S2B). It was not possible however to establish the chemical structure of these compounds.

In order to determine the number of sugars associated to the crocetin molecules, aqueous extracts were analysed by UPLC-QTOF-MS (Fig. S3). As shown in Table 1, most of the crocins are conjugated with 4–8 sugar moieties, and compounds with up to 8 glucose molecules were detected. In addition to a high level of homo and hetero-sugar conjugation, a new sugar was found in these crocins, a pentose, which was later identified as rhamnose by sugar analysis.

**Table 1:** Identification of crocins in *C. ancyrensis* tepals and stigmas by UPLC-UV/MS.

Peak number	Presence Tepals	Stigmas	*R*_t_ (min)	UV λ_max_ (nm)	Fragmentation pattern m/z	Tentative identification
1	√	√	1.02	446, 475	(ES+) 1647.00, 999.00, 587.00, 325.00	Trans-Crocetin +8 gluc
2	√	√	1.14	447, 469	(ES+) 1485.00, 1461.50, 1299.50, 999.36	Trans-Crocetin +7 gluc
3	√	√	1.32	445,470	(ES−) 1323.51, 649.14, 975.35	Trans-Crocetin +6 gluc
4	√		1.60	442, 468	(ES+) 1648.00, 1145.10, 827.23, 651.25, 489.20, 325.11	Trans-Crocetin +8 gluc
5	√		1.90	444, 462 (sh)	(ES+) 1297.00, 1135.37, 811.30, 487.16, 325.12	Trans-Crocetin +6gluc
6	√	√	1.99	328, 443, 468	(ES−) 1300.48, 975.36	Cis-Crocetin +6 gluc
7	√		2.23	327, 444, 468	(ES+) 1323.48, 1134.21	Cis-Crocetin +6 gluc
8	√	√	4.12	419	(ES−) 921.00, 624.00, 479.00	Trans-Crocetin +4 rham
9	√		4.36	328, 440, 470 (sh)	(ES+) 1297.44, 1135.37, 973.33, 811.28, 649.22, 487.17, 325.11(ES−) 1623.53	Cis-Crocetin +8 gluc
10		√	4.71	428	(ES−) 1123.45	Trans-Crocetin +4 rham
11	√	√	6.95	443, (sh)	(ES−) 975.35	Trans-Crocetin +4 Gluc
12		√	7.86	306, 414, 460	(ES−) 773.29	Cis-Crocetin +3 rham
13	√	√	8.31	328, 437, 470 (sh)	(ES−) 1299.5, 975.45, 324.10	Cis-Crocetin +6 gluc
14	√		8.45	438, 462	(ES−) 1136.00, 975.36	Trans-Crocetin +5 gluc
15			8.47	429, 445	(ES−) 961.00	Trans-Crocetin +3 gluc +1 rham
16	√	√	8.96	436, 461	(ES−) 1255.43, 1103.53, 813.00	Trans-Crocetin +3 gluc +2 rham
17		√	9.17	312, 429, 450 (sh)	(ES−) 1398.60, 973.29, 799.34	Cis-Crocetin +3 gluc +4 rham
18		√	9.35	312, 430	(ES−) 961.39, 647.20	Cis-Crocetin +3 gluc+1 rham
19		√	9.84	313, 418, 435 (sh)	(ES−) 959.37	Cis-Crocetin +3 gluc +1 rham
20		√	9.96	312, 431	(ES−) 959.37	Cis-Crocetin +3 gluc +1 rham
21		√	10.18	313, 424	(ES−) 961.40	Cis-Crocetin +3 gluc +1 rham
22	√	√	10.35	435, 460	(ES−) 551.20, 324.10	Trans-Crocetin +2 gluc
23	√	√	10.63	426, 440 (sh)	(ES−) 637.29, 324.10	Trans-Crocetin +1 gluc +1 rham

Data from negative and positive mode.

### Presence of crocins in the tepals of other spring-flowering Crocus species from the Series *Reticulati, Biflori, Orientales* and *Flavi*

**Figure 3:**
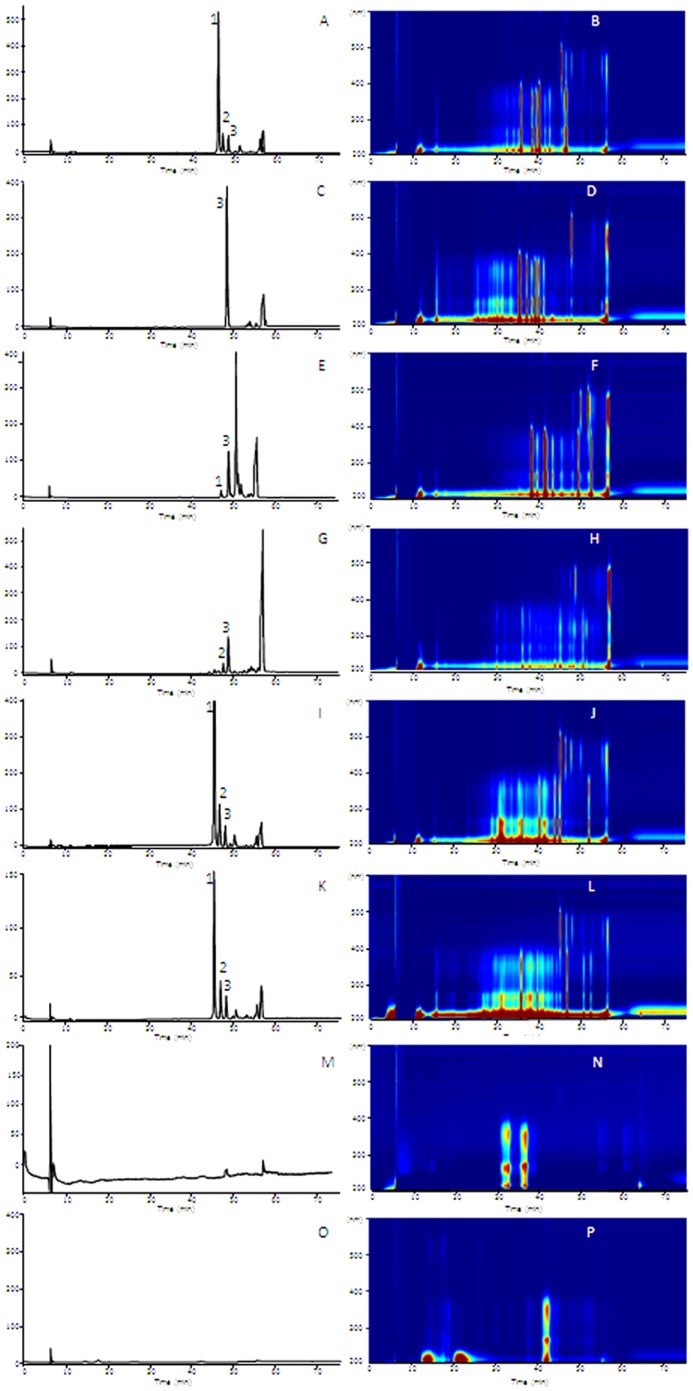
HPLC profile at 446-PDA/UV isoplot chromatogram of tepals aqueous extracts of different *Crocus* species with method A. *Crocus ancyrensis* (A, B), *C. angustifolius* (C, D), *C. chrysanthus* (E, F), *C. korolkowii* (G, H), *C. olivieri* (I, J), *C. vitellinus* (K, L), *C. sieberi* (M, N) and *C. sativus* (O, P).

**Figure 4:**
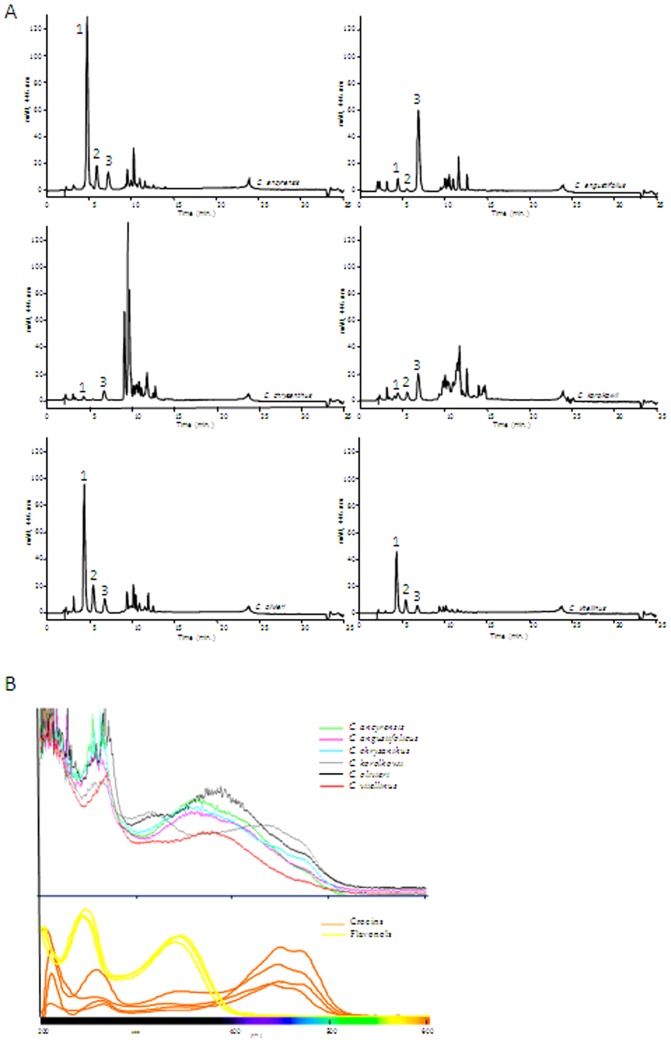
Representative HPLC chromatograms obtained with the chromatographic method B from aqueous extracts of tepals of *Crocus ancyrensis*, *C. angustifolius, C. chrysanthus*, *C. korolkowii*, *C. olivieri* and *C. vitellinus*. Labels correspond to compounds shown in Table 1.

The presence of the apocarotenoids crocetin, crocins and picrocrocin was also investigated in the tepals of *C. angustifolius*, *C. chrysanthus*, *C. olivieri*, *C. korolkowii*, *C. vitellinus* and *C. sieberi* (Fig. 1). With the exception of *C. sieberi*, which has a yellow patch at the base of the tepals, the other species showed yellow internal tepals and a yellow background colour in the external tepals. Two methods A and B were used in theses analyses of anthesis flower tepals. The chromatograms obtained with method A, which allows the determination of the flavonoid pattern and other metabolites in addition to crocins, were compared with those of *C. ancyrensis* (Fig. 3A and B) and *C. sativus* (Fig. 3O and P). Metabolites with a crocin-like spectra with maximum absorption around 446 nm were detected in *C. angustifolius* (Fig. 3C and D), *C. Chrysanthus* (Fig. 3E and F), C. *korollkowii* (Fig. 3G and H), *C. olivieri* (Fig. 3I and J), *C. vitellinus* (Fig. 3K and L) and in *C. sieberi* (Fig. 3M and N), whereas these metabolites were not detected in *C. sativus* (Fig. 3O and P), where only anthocyanins and flavonols glycosides were detected. Method B was used in order to obtain a higher resolution for the crocins present in the extracts (Fig. 4A). All the extracts were characterized by the presence of highly glycosylated crocins. Furthermore, in order to determine the contributions of these crocins to the yellow colour of these tepals, the full spectra of the different tepal extracts were compared with those from flavonoids and crocins, as major compounds observed in the extracts of tepals (Fig. 4B). The comparison showed that crocins were responsible for the observed yellow colour of tepals. This contribution was more evident in the case of *C. korolkowii*, which showed reduced levels of flavonoids (Fig. 3G and H).

The pattern of crocins in tepals of *C. ancyrensis*, *C. olivieri* and *C. vitellinus* contained highly glucosylated crocins and was very similar to that of *C. sieberi*, *C. chrysanthus* and *C. korolkowii* (Fig. 4, Table 2). The lowest levels of highly glucosylated crocins were detected in *C. chrysanthus*, where they reach only 1.55% of total absorbance at 446 nm in contrast to *C. vitellinus* (23%) and *C. ancyrensis* (22%). In general the observed levels of crocins (176.4–58 mAU (446 nm)/mg) (Table 2) were consistent with the presence and intensity of the yellow colour of the tepals of these species including the low levels of crocins in *C. sieberi* (5.2 mAU (446 nm)/mg), with a restricted colour location in the tepals, and with its complete absence in *C. sativus* (Fig. 1). The apocarotenoid picrocrocin, with a λmax at 250 nm, was not detected in any of the analysed spring *Crocus* species. Furthermore, carotenoid analysis of chloroform extracts only detected β-carotene whereas zeaxanthin was undetectable (data not shown).

**Table 2:** Levels of highly glucosylated crocins in stigmas and tepals of different *Crocus* species at anthesis.

*Crocus* species	Tissues	Fresh weight anthesis (mg)	mAU (446 nm)/mg fresh weight ×10^−3^			
			Peak 1	Peak 2	Peak 3	Total absorbance at 446 nm
*C. ancyrensis*	stigma	4.8±0.8	23±1.2	9.6±0.8	11.19±0.8	100±7.6
	tepal	18.3±1.4	24.48±1.6	3.57±1.4	3.02±0.7	142.22±8.1
*C. angustifolius*	stigma	9.4±2.1	n.d.	n.d.	115.9±3.6	316±4.8
	tepal	20.6±2	1.61±0.2	0.33±0.1	13.7±0.5	88.96±0.8
*C. chrysanthus*	stigma	7.8±0.23	n.d.	0.55±0.06	0.7±0.1	94.2±1.1
	tepal	23.03±1.8	0.35±0.03	0.15±0.005	1.4±0.2	122.05±2.2
*C. korolkowii*	stigma	4.9±0.9	41.2±1.6	13.9±0.8	20.3±1.2	104.5±3.1
	tepal	19.2±1.5	0.88±0.04	1.35±0.08	5.24±1.2	129.1±6.3
*C. olivieri*	stigma	4.9±0.9	55.1±4.5	7.54±1.6	7.1±2.0	92.06±4.8
	tepal	13.6±0.96	25.15±4.4	5.9±0.8	3.8±0.12	176.4±9.3
*C. sativus*	stigma	30±2.6	n.d.	n.d.	n.d.	n.d.
	tepal	39.3±1.9	n.d.	n.d.	n.d.	n.d.
*C. sieberi*	stigma	8.2±0.28	n.d.	n.d.	28.6±1.8	120.9±6.3
	tepal	31.8±2.8	n.d.	n.d.	2.47±0.15	1.35±0.2
		yellow section: 9.33±0.95	0.46±0.08	0.61±0.044	2.73±0.06	5.2±2.2
*C. vitellinus*	stigma	4.8±0.8	30.25±4.6	5.72±2.3	11.52±2.7	91.8±5.3
	tepal	18.6±1.3	8.43±1.5	2.5±0.06	1.42±0.08	53.8±3.4

### Highly glycosylated crocins also accumulate in the stigma of *C. ancyrensis*

**Figure 5:**
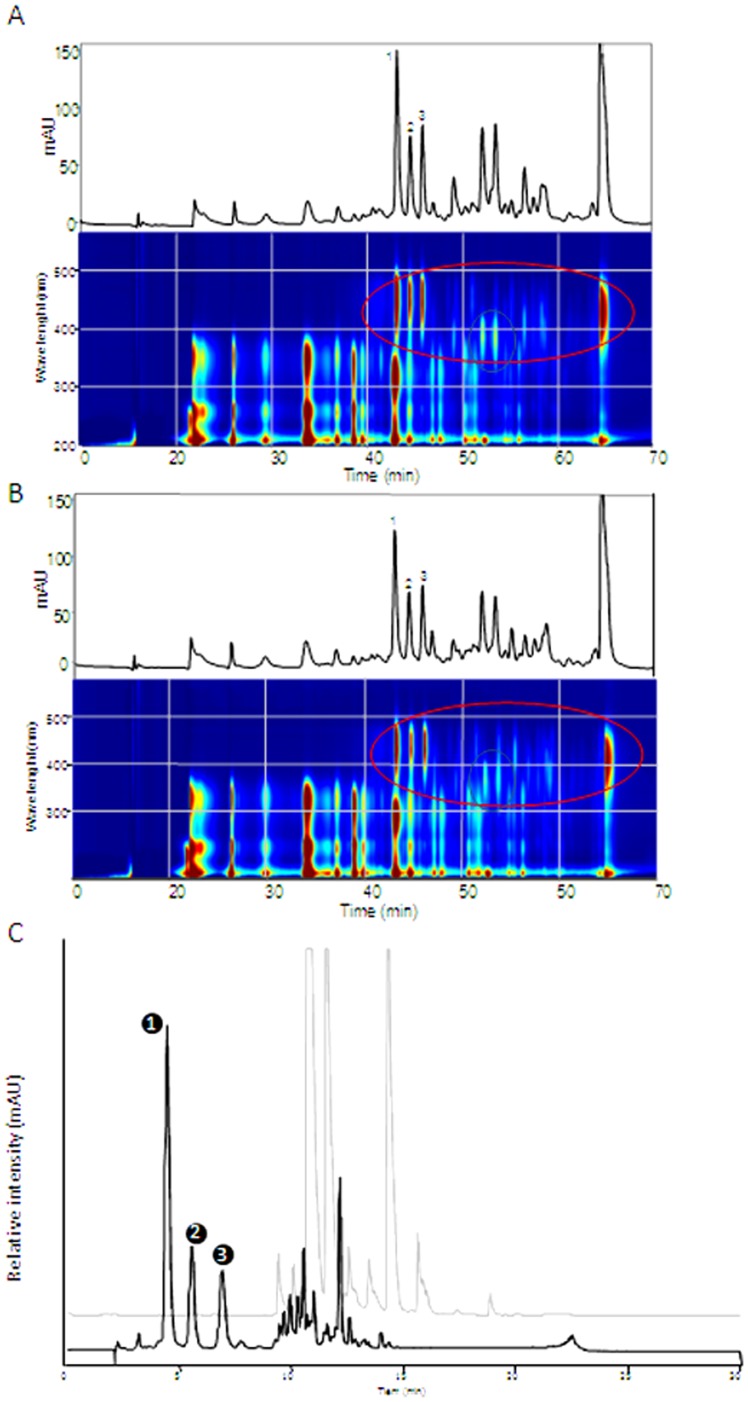
Heatmap showing changes of crocins levels comparing stigmas with tepals. Red indicates increased metabolite levels, and blue represents decreased levels (see colour scale bar).

The yellow to red colour observed in the stigmas of *Crocus* species correlates with the accumulation of crocins in that tissue [16], [31]. Preanthesis stigmas and stigmas at anthesis from *C. ancyrensis* were analyzed as previously described [16] for the presence of crocins and picrocrocin. The stigmas at both stages showed a very similar chromatographic profile (Fig. 5A and B). In addition to the presence of glycosylated flavonoids (detected at 250–350 nm), several compounds (Fig. 5A and B) with absorption maxima and UV spectra resembling those or crocins and crocetin (C20; absorption maxima between 424 and 446 nm) and C_14_ apocarotenoid (absorption maxima between 379 and 399 nm) [42] were detected. The profile of stigma crocins obtained for *C. ancyrensis* was compared with the one obtained for *C. sativus* by means of the method B, which is used for routine analysis of crocins in saffron (Fig. 5C). Although crocins are present at more reduced levels in *C. ancyrensis* stigmas, the retention time of the identified crocins suggested a more glycosylated pattern of crocins than that observed in *C. sativus* (Fig. 5C).

In order to investigate in more detail what apocarotenoids in addition to crocetin could be present in stigmas as glycosylated forms, both basic and acid hydrolysis were performed on the aqueous extract followed by UPLC-MS (Fig. S4). In the case of acid hydrolysis two main compounds (1 and 3) and three minor compounds (2, 4 and 5) were detected at 446 nm. These compounds could not be detected before hydrolysis and were tentatively identified as crocetin isomers [18]: compound 1 (rt. 14.93; λ_max_ trans- 405, 427, 454 nm; m/z 625.32 (2 crocetin), 325.18), compound 2 (rt. 17.30; λ_max_ cis- 315, 405, 422, 448 nm; m/z 329.18 (M+), 327.10 (M−) (crocetin), 311.16 (crocetin -H_2_O), compound 3 (rt. 18.62; λ_max_ trans- 405, 427, 454 nm; m/z 311.17 (crocetin - H_2_O), 293), compound 4 (rt. 19.80; λ_max_ Cis- 315.3, 405, 422, 448 nm; m/z 325.18 (crocetin)) and compound 5 (rt. 21.60; λ_max_ trans- 412, 427, 453 nm; m/z 325.18 (crocetin)). Alkaline hydrolysis on the same extracts resulted in a reduced number of crocetin isomers and in the detection of two main crocetin forms trans-crocetin and cis-crocetin (Fig. S4C).

In addition to crocetin, four additional compounds were detected at 400 nm after hydrolysis of the stigma extracts and tentatively identified as different cis- isomers of the acyclic C_14_ polyene 10,10′-diapocarotene-10,10′-dioic acid, (C_14_) [36], [43]. These include: compound I (rt. 11.32; λ_max_ 277, 380, 399 nm; m/z ES+ 555.00, 278.03), compound II (rt. 12.71; λ_max_ 278, 379, 398 nm; m/z ES+ 563.00, 278.03), compound III (rt. 14.14; λ_max_ 285, 388, 400 nm) and compound IV (rt. 15.08; λ_max_ 279, 379, 398 nm; m/z ES+ 278.01) (Fig. 4A). Analysis of the extracts before or after hydrolysis did not reveal the presence of HTCC, the saffron apocarotenoid precursor of picrocrocin.

In order to determine the molecular composition of the crocins observed in the stigma tissue, the aqueous extract was analysed by UPLC-Q TOF MS under conditions that produced discrete molecule fragmentation (Fig. S5). As shown in Table 1, crocins with up to 8 glucose molecules were also detected in the stigma, as well as crocins with rhamnose. Table 1 shows that the major crocin-like chromatographic peaks were common between stigmas and tepals, but specific crocins were also observed to characterize either stigmas and tepals (Fig. S3 and S5). Moreover, it was generally observed that the stigma profile was much more complex than the one obtained for tepals, and it also contained a higher number of glucosylated apocarotenoids, including apocarotenoids different to crocetin.

### Crocins in the stigmas of the spring Crocus species

The crocins pattern of several autumn *Crocus* stigmas had been analysed previously [16], but only one study has been performed on one spring-flowering species [44]. The detection of crocins with different glucosylation profiles in the tepals of these species, prompted us to determine whether crocins follow a similar pattern in the stigma tissue. As the crocin profile could change during development [17], samples corresponding to preanthesis and anthesis stages were selected for analysis. The crocin profile in preanthesis and anthesis stigmas of *C. angustifolius*, *C. chrysanthus*, *C. olivieri*, *C. korolkowii* and *C. vitellinus* was obtained and compared to that of stigmas of *C. ancyrensis* at preanthesis and anthesis, and with the stigmas from *C. sieberi* and *C. sativus* at anthesis (Fig. S6).

**Figure 6:**
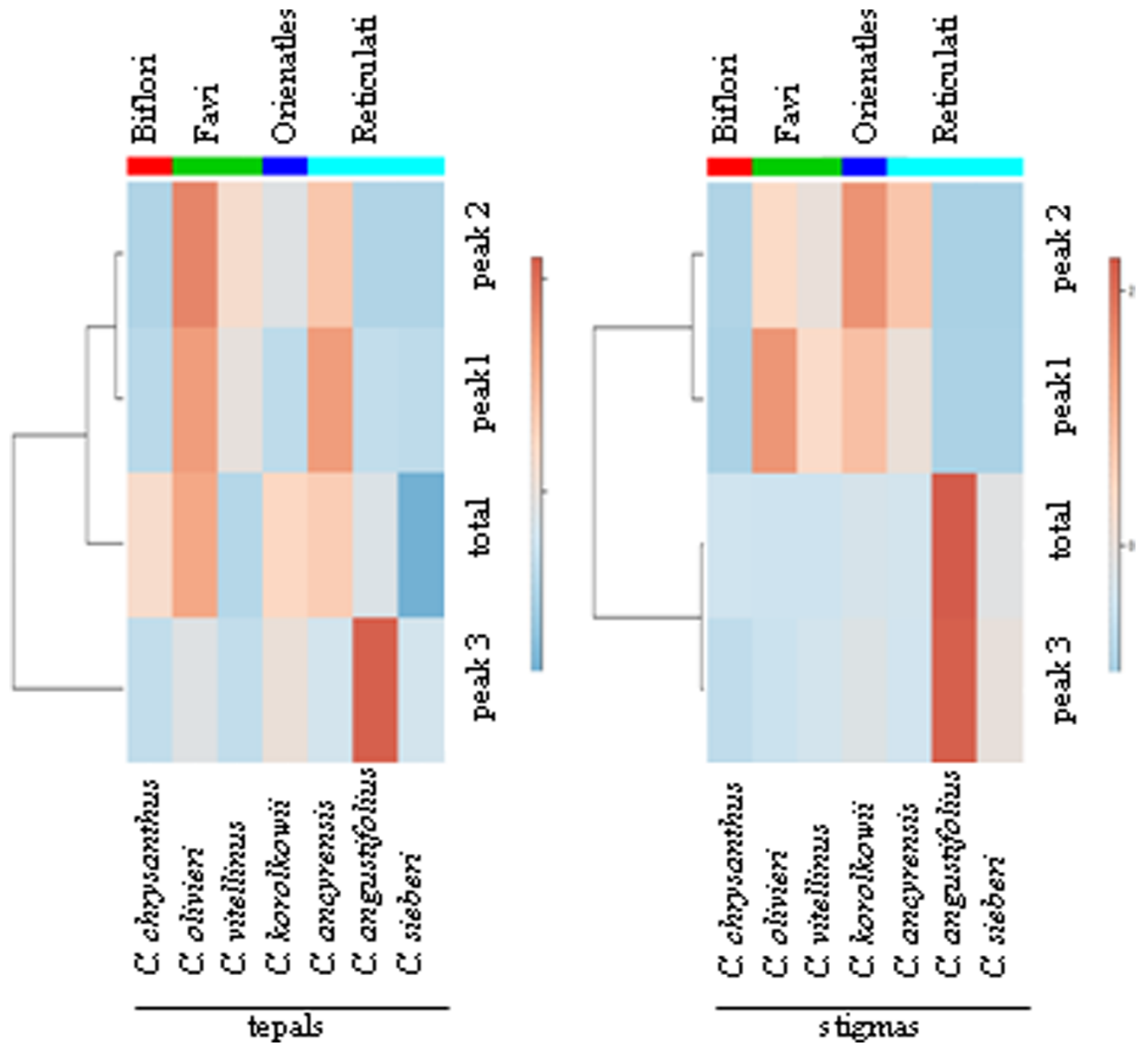
HPLC-PDA chromatograms of aqueous extracts of stigmas in preanthesis and in anthesis of *Crocus ancyrensis*, *C. angustifolius*, *C. chrysanthus*, *C. korolkowii*, *C. olivieri* and *C. vitellinus*. For *C. sieberi* and *C. sativus* only anthesis chromatograms are shown. Labels correspond to compounds shown in Table 1.

In order to present a global view of the obtained dataset, the results were visualized with a heatmap. The results of the analyses showed a clear distinctive profile for stigmas compared to tepal tissue (Fig. 6). Further, the same type of tissues from the species that belong to the same series also exhibited differentiation (Fig. 6) in the majority of the samples, except for the stigmas of *C. olivieri* and *C. vitellinus*, whose patterns were in agreement with the presence of both species in the same series (Fig. 6).

### Expression analysis of genes related with crocin biosynthesis in tepals

**Figure 7:**
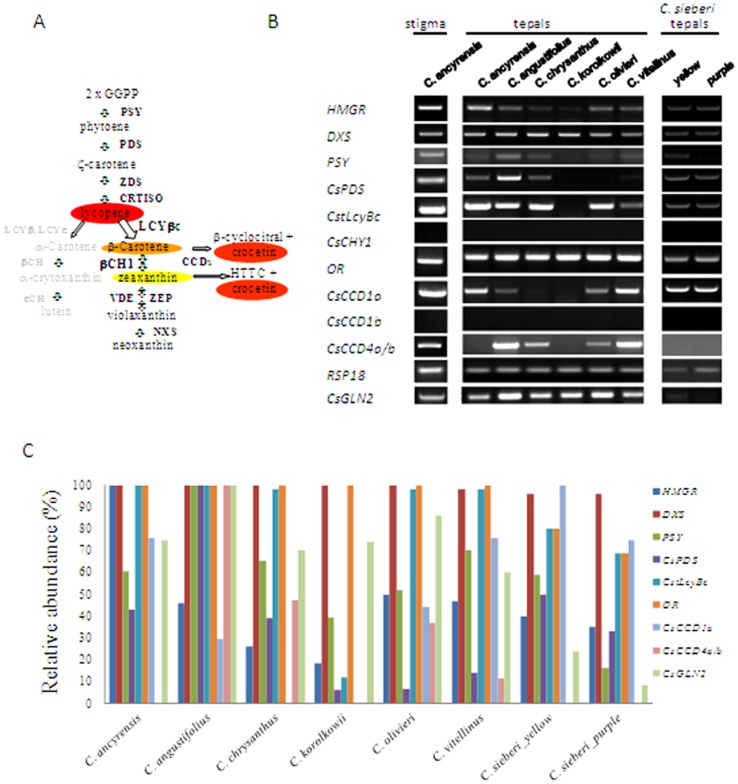
Expression analysis of selected genes involved in carotenoid metabolism. A. Schematic carotenoid and apocarotenoid biosynthetic pathway in *Crocus*. Enzymatic reactions are represented by arrows. GGPP, geranyl geranyl diphosphate; PSY, phytoene synthase; PDS, phytoene desaturase; ZDS, z-carotene desaturase; CRTISO, carotene isomerase; LCYe, lycopene e-cyclase; LCYb, lycopene b-cyclase; bCH, b-carotene hydroxylase; eCH, e-carotene hydroxylase; ZEP, zeaxanthin epoxidase; VDE, violaxanthin de-epoxidase; NXS, neoxanthin synthase; CCD, carotenoid cleavage dioxygenase. B. RT-PCR analysis for expression of selected candidate genes related to the carotenoid metabolism. Equal amounts of total RNA were used in each reaction. The levels of constitutively expressed *RPS18* coding gene were assayed as controls. The PCR products were separated by 1.5% (w/v) agarose gel electrophoresis and visualized by ethidium bromide staining. C. Relative transcript levels in tepals of the genes analysed by RT-PCR in B.

To gain insight into the relative importance of the different enzymatic steps leading to crocin in tepals, a targeted gene expression analysis was performed. In our approach the relative transcript levels of two genes involved in general isoprenoid (including carotenoids) metabolism 1-deoxy-d-xylulose 5-phosphate synthase (DXS), the first enzyme specific to the MEP pathway, and 3-hydroxy-3-methylglutaryl CoA reductase, HMGR, the enzyme that catalysed the third step of the mevalonate (MVA) pathway in the cytoplasm, along with three genes involved in carotenoid biosynthesis (Fig. 7A): phytoene synthase (PSY), phytoene desaturase (CsPDS), the lycopene β-cyclase (CstLycBc) and the carotene β-hydroxylase (CsCHY1) were analysed. In addition, the expression levels of the *Or* gene involved in carotenoid accumulation [45] and genes encoding for enzymes involved in carotenoid cleavage in saffron [46] such as *CsCCD1a*, *CsCCD1b* and *CsCCD4a/b*, were measured in stigmas and tepals from *C. ancyrensis*, and in tepals of *C. angustifolius*, *C. chrysanthus*, *C. olivieri*, *C. korolkowii*, *C. vitellinus* and *C. sieberi*, by reverse transcription semi-quantitative PCR (RT-PCR) (Fig. 7B and C).

The isoprenoid pathway gene, *DXS* showed relatively high transcript levels in all the analysed samples (Fig. 7B and C). By contrast, the *HMGR* gene showed different transcript levels, compared to *DXS*, in all the samples. The early carotenoid pathway genes *CsPSY* and *CsPDS* showed relatively high transcript levels in *C. ancyrensis* stigmas and tepals, with similar levels in the yellow sectors of *C. sieberi* tepals, and with the highest levels in *C. angustifolius* tepals. *PSY* transcripts were also detected at lower levels in *C. vitellinus*, *C. olivieri* and *C. korolkowii*, and were undetectable in purple sectors of *C. sieberi* tepals. However, *CsPDS* expression levels were below detection level in *C. korolkowii* and *C. olivieri* (Fig. 7B and C) by only five cycles in our RT-PCR reactions (data not shown). The *CsCHY1* gene is a chromoplast-specific lycopene beta cyclase gene involved in the high production of saffron's apocarotenoid precursors in the stigma tissue of *C. sativus* [31]. This gene was highly expressed in *C. ancyrensis*, *C. angustifolius*, *C. chrysanthus*, *C. olivieri*, *C. sieberi* and *C. vitellinus* and expressed at very low levels in *C. korolkowii* tepals (Fig. 7B and C). *CsCHY1* transcripts were not detected in any of the samples, as expected by the absence of zeaxanthin, picrocrocin and its precursor HTCC, generated from the cleavage of zeaxanthin. The transcript levels of the *Or* gene, which acts as a bona fide molecular switch to trigger the differentiation of non-coloured plastids into chromoplasts [45], were present at high levels in all the analysed samples (Fig. 7B and C), with more reduced levels in the purple sectors of *C. sieberi* tepals.

Carotenoid catabolism produces diverse apocarotenoid compounds which confer fruits and flowers their characteristic aromas and colours and contribute to attracting pollinators and seed dispersers. Such apocarotenoids are produced when carotenoids are cleaved by CCDs. Gene expression profiles of *CsCCD1a*, *CsCCD1b* and *CsCCD4a/b* were examined (Fig. 7B and C). *CsCCD1a* was detected in all the samples, except in *C. korolkowii*, with the highest levels in the yellow sectors of *C. sieberi* tepals followed by the levels in *C. ancyrensis* stigmas and tepals, purple sectors of *C. sieberi* tepals and *C. vitellinus* tepals (Fig. 7B and C). *CsCCD1b* transcript levels were undetectable in all the analysed samples (Fig. 7B and C). In *C. sativus*, *CsCCD1b* has been only detected in the earliest developmental stages of the stigma [46]. The expression of *CsCCD4a/b*, highly expressed in the late developmental stages in *C. sativus* stigmas and at lower levels in tepals [46], was significantly different among the analysed samples, with high levels of expression in the tepals of *C. angustifolius*, followed by *C. vitellinus* and C. *ancyrensis* stigmas, and lower levels in the tepals of *C. chrysanthus* and *C. olivieri*, while being completely absent from the tepals of *C. ancyrensis*, *C. sieberi* and *C. korolkowii* (Fig. 7B and C). The high expression of *CsCCD4a/b* in the stigmas of *C. ancyrensis*, could explain the accumulation in this tissue of 10,10′-diapocantoene-10,10′-dioic acid, not detected in tepals.

Once crocetin is generated by oxidative cleavage, it becomes a substrate for glucosyltransferases to render crocins. Previous studies indicated that at least 2 glucosyltransferases from saffron are involved in the glucosylation of crocetin and crocin esters in the stigma tissue [47]. CsGLN2 catalyzes the ester bond formation between the carboxyl residues of crocetin or crocin and the glucosyl moiety of UDP-glucose [19]. Due to the presence of highly glucosylated crocins in tepals, the expression of *CsGLT2* was analysed in these tissues (Fig. 7B and C). *CsGLN2* transcripts were detected in all the analysed samples with the highest levels of expression in *C. angustifolius*, which contains the highest levels of total soluble apocarotenoids at 446 nm, and the lowest levels of expression in *C. sieberi* tepals, which contains the lowest levels of these apocarotenoids.

## Discussion

In the present study, we have used a combination of approaches to determine the pigments responsible for the yellow colour observed in *Crocus* flowers from the *Reticulati*, *Orientales, Biflori* and *Flavi* Series from the *Nudiscapus* section. The tepals of the species in these sections showed a variety of colours (Table S1). All the species with yellow tepals are winter-to-early spring flowering, and no yellow flowers are found among *Crocus* flowering in autumn. We describe here that the observed yellow color is due to the presence of crocins and among them, highly glucosylated crocins that have never been described before in autumn or spring species, contribute to this colour.

The petals of some plants have introduced modifications in the carotenoid biosynthetic pathways to produce carotenoid compositions that characterize their respective genus or species. Some plant petals exhibiting unique colours such as yellow, orange and red are due to the presence of apocarotenoids, which also contribute to flower scent [2]. For instance, the petals of *Eschscholzia californica* (Papaveraceae) accumulate crocetin among other carotenoids [48] and those of *Crocosmia* (Iridaceae) accumulate crocin [49]. Various apocarotenoids such as β-ionone, 10′-apocaroten-10′-oic acid, and hydroxy 10′-apocaroten- 10′-oic acid are found in the petals of *Boronia megastigma* [50].

By using high-performance liquid chromatography, coupled with photodiode array detection and electrospray ionization mass spectrometry, we have found that crocins accumulated at relatively high levels in the tepals of *C. ancyrensis, C. angustifolius, C. olivieri, C. korolkowii* and *C. vitellinus*, where they contribute to the yellow colour of the tepals in these species. These crocins showed higher glucosylation levels in comparison to the crocins previously described in *C. sativus* stigmas and in fruits of *Gardenia jasminoides* where crocins with up to five glucose molecules have been identified [51]. However, crocetin with six glucose molecules (crocetin di-neapolitanosyl ester) have been obtained as the main crocin in cell cultures of saffron supplemented with crocetin [52]. Here crocins with up to 8 sugar molecules were observed in tepals of *C. ancyrensis, C. angustifolius, C. olivieri, C. korolkowii*, C. *vitellinus* and *C. sieberi*. But also crocins with 7 and 6 glucose molecules were identified. In addition, these highly glucosylated crocins were the main crocins detected in the tepals of the yellow crocuses. In *C. sativus*, glucosylation of crocetin into crocin is sequential and might involve two distinct glucosyltransferases [47]. A glucosyltransferase involved in crocin glucosylation, CsGLN2, was isolated and characterized from *C. sativus* stigmas [19]. This enzyme is able to add between 9 to 18 glucose molecules to crocetin *in vitro*, which suggests that it could perform the same activity *in vivo*, at least in the yellow tepals and stigmas of the *Crocus* species analyzed in this study.

Differences in retention times for highly glucosylated crocins with the same number of glucose molecules, suggest a different arrangements of the glucose molecules on each of the ends of the crocetin molecule [35]. Highly glucosylated crocins were also detected in the stigma tissue of these spring species, although the crocins profiles were different between the stigmas and tepals of the same species. Although it was not possible to establish a relationship between the pattern of highly glucosylated crocins and species belonging to the same series, the presence of these crocins in the stigmas seems to be characteristic of spring crocuses in contrast with their absence in the stigma of autumn crocuses [16]. It is not easy to find a reason for the presence of highly glucosylated crocins in the spring-crocus species, and previous studies have shown that glucosyltransferase activity does not seem to be a limitation [19]. In contrast to autumn species, the leaves in spring species develop much more in advance than flowers do and hence more carbon can be fixed photosynthetically [53]. The availability of enough glucose could favour the formation of these highly glucosylated crocins, as has been observed in saffron cell cultures where crocetin with six glucose molecules is the main crocin present [52].

The detailed analysis of crocins in the stigmas and tepals of *C. ancyrensis* showed the conjugation of rhamnose molecules in addition to glucose not described before in crocins, suggesting the presence of a new glycosyltransferase activity in this species. Rhamnose has been shown though to be present in other carotenoid and apocarotenoid glycosides [54], [55]. The stigmas of *C. ancyrensis* were also characterized by the presence of additional apocarotenoids different from crocin which in addition were absent in the tepals. These apocarotenoids were also detected in the stigmas of *C. angustifolius* and C. *korolkowii* but not in the stigma of the other analysed species. The carotenoids β-carotene and zeaxanthin act as precursors of crocetin and crocins in *C. sativus* stigmas [17]. In *C. sativus* these pigments are restricted to the stigma tissue, and are absent from the other flower tissues. However, this restriction is not found in *C. ancyrensis*, *C. angustifolius*, *C. olivieri*, *C. korolkowii*, *C. vitellinus*, and *C. sieberi*, suggesting a different regulatory control on crocetin and crocin biosynthesis in these species. In *C. sativus* and in other *Crocus* species flowering in autumn, the reactions catalysed by CstLcyBc and CHY1Cs enzymes play a key role in the formation of saffron apocarotenoids in the stigma tissue, due to the accumulation of the respective substrates and products in the stigmas [16], [31]. Nevertheless, the levels of carotenoids in the developed stigmas are much lower as compared with the massive accumulation of apocarotenoids, suggesting a high flux rate in the carotenoid pathway and an important role for the carotenoid cleavage dioxygenases [56]. We analysed the expression levels of *CstLcyBc* and *CHY1Cs* in these *Crocus* with yellow tepals. *CHY1Cs* was not detected in any of the analysed species, which is in agreement with the absence of zeaxanthin in the stigmas and tepals of these species, and also with the absence of picrocrocin. The levels of expression of *CstLcyBc* correlate with the presence of β-carotene in the tepals of all these species, but not with the levels of crocins in the tissues of these species. We cannot exclude however that crocin levels could be associated to the total levels of all the apocarotenoids, not just crocins, which could be derived from β-carotene, including volatile compounds. If so, the CCDs enzymes should play a key role in the accumulation of their respective apocarotenoid products. Furthermore, other apocarotenoids, tentatively identified as isomers of 10,10′-diapocarotenedioic acid (C_14_) and present at lower levels, were found to be present in the stigma and as crocetin, in glycosylated form. These compounds were also detected in the stigma of *C. korolkowii*, and at higher levels in the stigmas of *C. angustifolius* (Fig. S7), but not detected in tepals. Interestingly, such compounds had never been identified before in the flowers of the genus *Crocus*, including *C. sativus*, although the stigma of *C. sativus* produces significant levels of β-ionone at anthesis [17], suggesting a fast turnover for this C_14_ apocarotenoid in this tissue by the action of CCDs, which are highly expressed in *C. sativus* stigmas [46].

The activity of CCDs could explain the relative low levels of total crocins detected in the tepals of *C. angustifolius* and *C. vitellinus*. In these species the carotenogenic genes were relatively highly expressed, which suggested the presence of high production of carotenoids, as substrates for crocetin formation. In addition, high levels of *CsCCD4a/b* and *CsCCD1* were observed. CsCCD4a/b cleave carotenoids at the 9,10 (9′,10′) double bonds [46], and CCD1 enzymes cleave a variety of carotenoids at the 9,10 (9′,10′) double bonds [57] although recent studies suggest that apocarotenoids instead of carotenoids act as the major substrates of CCD1 in the plant [41], [58]. Therefore, CCD1 could acts in these cases against crocetin accumulation either by acting on the substrates for crocetin formation as CCD4 enzymes do, or directly by the cleavage of this apocarotenoid. Furthermore, the apocarotenoids generated by CCD activities could act as activators of the genes of the carotenoid pathway, as has been proposed in fungi [59] and in tomato [60]. This feedback mechanism might explain the higher levels of *PSY* and *CsPDS* observed for *C. angustifolius* in comparison with *C. olivieri*, which accumulates the highest levels of crocins despite its low levels of *PSY* and *CsPDS*. In addition, the CCD enzyme responsible for crocetin formation still remains unknown [46], [61], and is probably expressed at low levels in these *C. angustifolius* and *C. olivieri* species. Moreover, the same could be the case for *C. sieberi* tepals, which would explain the low levels of crocin in this tissue independently of its local distribution.

In plants, the presence of crocins in flowers and fruits suggests their involvement in pollinator attraction and in seed dispersal. For humans crocins are much more than just a hydrosoluble natural yellow food colorant. Modern pharmacological studies have demonstrated that crocins possess anti-tumour effects, display anti-inflammatory properties, counteract atherosclerosis and hepatic damage and affect a number of different neural processes [62], [63]. Currently, the discovery of new sources of crocins is of major interest due to the high market price. Furthermore, glucosylation may improve the stability, activity, biological availability, and water solubility of molecules with pharmaceutical or nutritional interest [64]. In fact, highly glycosylated crocins seem to be more effective for the treatment of certain diseases [65]. Thus, the new crocins identified in this study, as characteristic pigments of yellow spring-flowering species, could potentially act as effective agents for the treatment of several human diseases.

## Supporting Information

Figure S1 The glucosylated apocarotenoids picocrocin and crocetin accumulate at high levels in the stigmas of *C. sativus*. Crocin formation involves the stepwise addition of a glucose moiety at the free end of the crocetin molecule and the 1→6 addition of a glucose moiety to the glucosyl ester function.
(TIF)

Figure S2 Analysis of the tepal extracts of *C. ancyrensis* after hydrolysis treatment. A, Typical chromatogram at 446 nm of tepal extracts of *C. ancyrensis* after alcaline hydrolysis. Inset are shown the absorbance spectra of trans-crocetin and cis-crocetin, respectively. B, Typical chromatogram at 310 nm obtained from reversed-phase LC-PDA-ESI-QTOF-MS analysis of *C. ancyrensis* tepal extracts hydrolyzed with HCl. Retention times (in minutes) are indicated for the most intense peaks. Inserts show absorbance spectrum (I) and MS/MS spectrum (II), for the unidentified compounds eluting at 1.66, 2.131 and 2.51 min.
(TIF)

Figure S3 Typical chromatograms obtained from reversed-phase LC-PDA-ESI-QTOF-MS analysis of *C. ancyrensis* tepal extracts at 442 nm. Retention times (in minutes) are indicated for the most intense peaks. B is a magnification from minute 1.4 from A. Inserts in B show absorbance spectrum (I) and MS/MS spectrum (II), for the compound eluting at 4.36 min, identified as crocetin +6 glucose molecules. C Absorbance spectra and MS/MS spectra of compounds eluting in A at 1.02, 1.14 and 1.25 min, identified as trans-crocetin with 8 glucose molecules, trans-crocetin with seven glucose molecules and trans-crocetin with six glucose molecules.
(TIF)

Figure S4 Hydrolysis of the aqueouse extracts of *C. ancyrensis* stigma. Chromatograms obtained from reversed-phase LC-PDA-ESI-QTOF-MS analysis of C. ancyrensis hydrolyzed stigma extracts at 390 (A) and at 442 nm (B). The absorbance spectrum is shown for the flavonoid kaempferol (K) and for the apocarotenoids 10,10′-diapocarotene-10,10′-dioic acid, (C14) (I) and 8,8′-diapocarotenedioic- 8,8′-dioic acid (crocetin) (1). C. LC-DAD chromatogram at 446 nm obtained with Method B over hydrolyzed extracts of stigmas of *C. ancyrensis* using NaOH. Inset are shown the absorbance spectra of peaks 1 and 3.
(TIF)

Figure S5 Typical chromatograms obtained from reversed-phase LC-PDA-ESI-QTOF-MS analysis of *C. ancyrensis* tepal extracts at 442 nm. Retention times (in minutes) are indicated for the most intense peaks. A. Inserts in A show absorbance spectra and MS/MS spectra for the compound eluting at 8.96 min, tentatively identified as Trans-Crocetin +3 glucose +2 rhamnose. B is a magnification of chromatogram A after1.5 min. Inserts in B show absorbance spectrum (I) and MS/MS spectrum (II), for the compound eluting at 4.36 min, identified as cis-crocetin +6 glucose molecules.
(TIF)

Figure S6 Presence of apocarotenoids in *C. ancyrensis* stigmas. A, In the upper part the HPLC profile of preanthesis stigma at 400 nm and below LC-PDA/UV isoplot chromatogram showing the compounds detected (200–550 nm). B, In the upper part the HPLC profile of anthesis stigma at 400 nm and below LC-PDA/UV isoplot chromatogram showing all the compounds detected (200–550 nm). C, Representative HPLC chromatograms from aqueous extracts of *C. ancyrensis* stigma (black line) and *C. sativus* stigma (grey line) at 446 nm. Labels correspond to compounds shown in Table 1. The red and grey circles denoted the compounds with absorbance between 424–446 nm and 379–399 nm, respectively.
(TIF)

Figure S7 Presence of different types of apocarotenoids in the stigma extracts of several *Crocus* species. A. LC-PDA/UV isoplot chromatogram showing all the compounds detected (200–550 nm) of stigma aqueous extracts of different *Crocus* species with chromatographic method A. The red circles denoted the compounds with absorbance between 379–399. B. Absorbance spectrum of non-crocin apocarotenoids detected in the stigmas of *C. ancyrensis*.
(TIF)

Table S1 Flowering time, tepal color and distribution of *Crocus* species from section Nudiscapus. The most recent classification is from Mathew (Mathew, 1982). He divided section Nudiscapus in nine series based on the division of the style, corm tunic features and flowering time. *Crocus* species with yellow tepals are highlight in yellow.
(DOC)
