# Supplementary figures and images for: Crocins with High Levels of Sugar Conjugation Contribute to the Yellow Colours of Early-Spring Flowering Crocus Tepals
Source: PLoS One. 2013 Sep 13;8(9):e71946. doi: 10.1371/journal.pone.0071946 (PMC3772802; doi:10.1371/journal.pone.0071946)

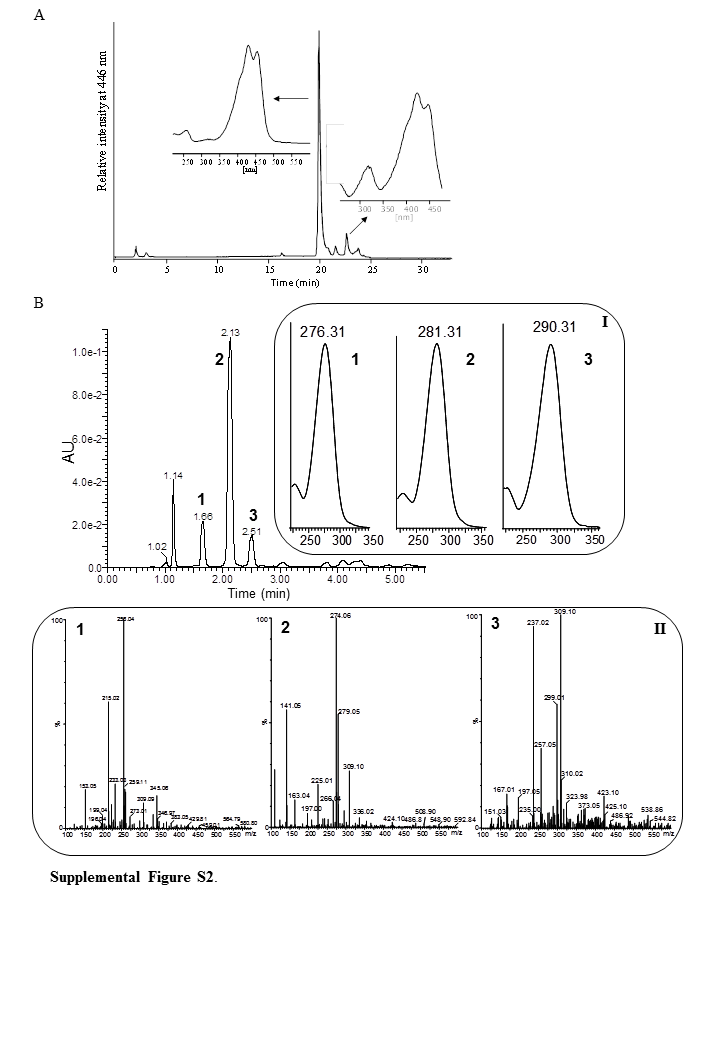

Supplement: Figure S2 — Analysis of the tepal extracts of C. ancyrensis after hydrolysis treatment. A, Typical chromatogram at 446 nm of tepal extracts of C. ancyrensis after alcaline hydrolysis. Inset are shown the absorbance spectra of trans-crocetin and cis-crocetin, respectively. B, Typical chromatogram at 310 nm obtained from reversed-phase LC-PDA-ESI-QTOF-MS analysis of C. ancyrensis tepal extracts hydrolyzed with HCl. Retention times (in minutes) are indicated for the most intense peaks. Inserts show absorbance spectrum (I) and MS/MS spectrum (II), for the unidentified compounds eluting at 1.66, 2.131 and 2.51 min. (TIF) [file pone.0071946.s002.tif]

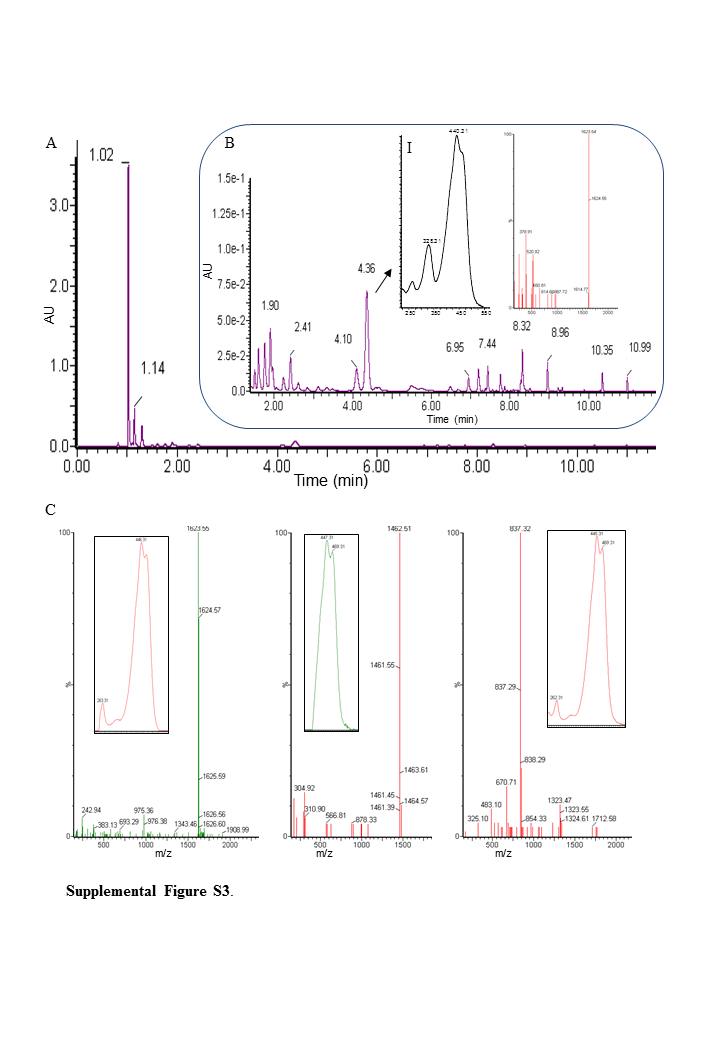

Supplement: Figure S3 — Typical chromatograms obtained from reversed-phase LC-PDA-ESI-QTOF-MS analysis of C. ancyrensis tepal extracts at 442 nm. Retention times (in minutes) are indicated for the most intense peaks. B is a magnification from minute 1.4 from A. Inserts in B show absorbance spectrum (I) and MS/MS spectrum (II), for the compound eluting at 4.36 min, identified as crocetin +6 glucose molecules. C Absorbance spectra and MS/MS spectra of compounds eluting in A at 1.02, 1.14 and 1.25 min, identified as trans-crocetin with 8 glucose molecules, trans-crocetin with seven glucose molecules and trans-crocetin with six glucose molecules. (TIF) [file pone.0071946.s003.tif]

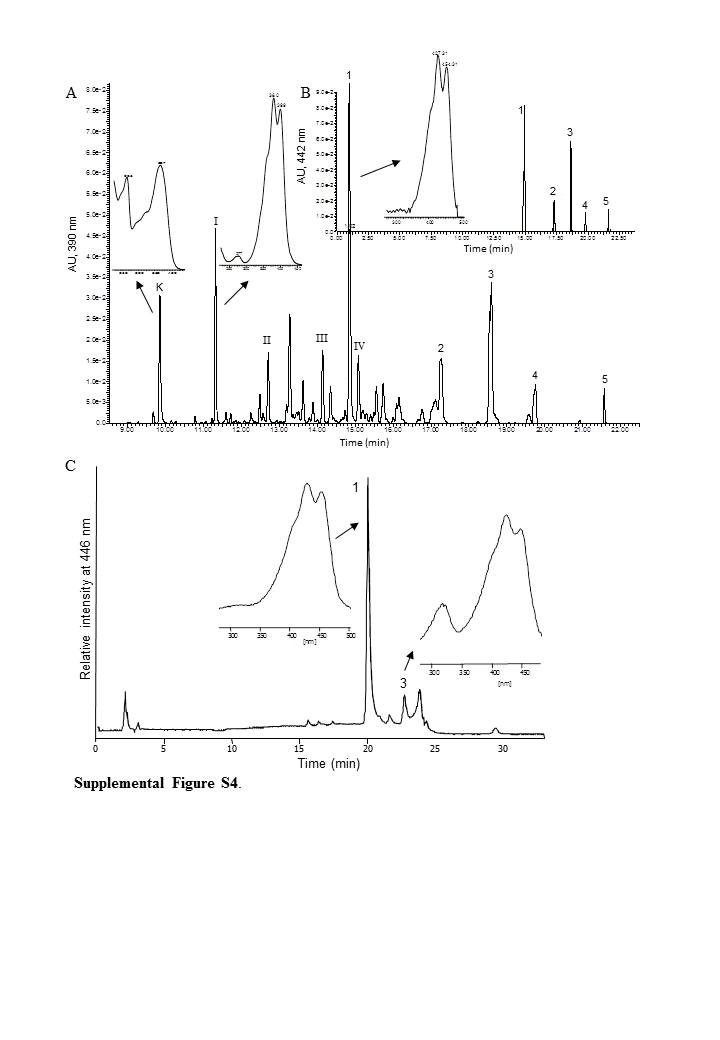

Supplement: Figure S4 — Hydrolysis of the aqueouse extracts of C. ancyrensis stigma. Chromatograms obtained from reversed-phase LC-PDA-ESI-QTOF-MS analysis of C. ancyrensis hydrolyzed stigma extracts at 390 (A) and at 442 nm (B). The absorbance spectrum is shown for the flavonoid kaempferol (K) and for the apocarotenoids 10,10′-diapocarotene-10,10′-dioic acid, (C14) (I) and 8,8′-diapocarotenedioic- 8,8′-dioic acid (crocetin) (1). C. LC-DAD chromatogram at 446 nm obtained with Method B over hydrolyzed extracts of stigmas of C. ancyrensis using NaOH. Inset are shown the absorbance spectra of peaks 1 and 3. (TIF) [file pone.0071946.s004.tif]

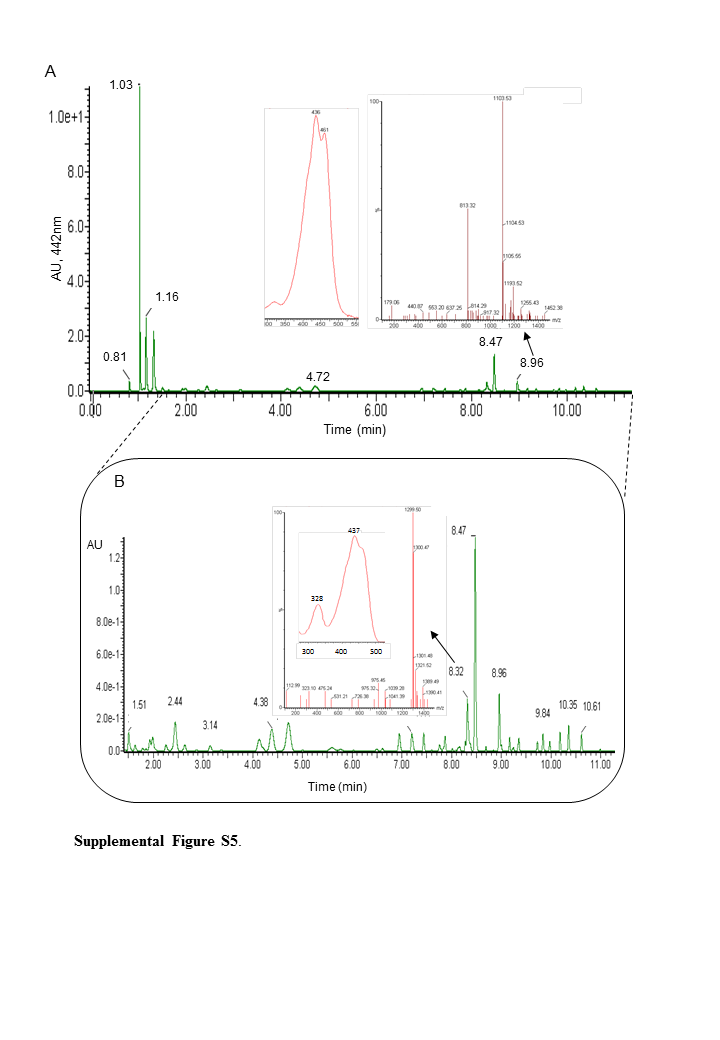

Supplement: Figure S5 — Typical chromatograms obtained from reversed-phase LC-PDA-ESI-QTOF-MS analysis of C. ancyrensis tepal extracts at 442 nm. Retention times (in minutes) are indicated for the most intense peaks. A. Inserts in A show absorbance spectra and MS/MS spectra for the compound eluting at 8.96 min, tentatively identified as Trans-Crocetin +3 glucose +2 rhamnose. B is a magnification of chromatogram A after1.5 min. Inserts in B show absorbance spectrum (I) and MS/MS spectrum (II), for the compound eluting at 4.36 min, identified as cis-crocetin +6 glucose molecules. (TIF) [file pone.0071946.s005.tif]

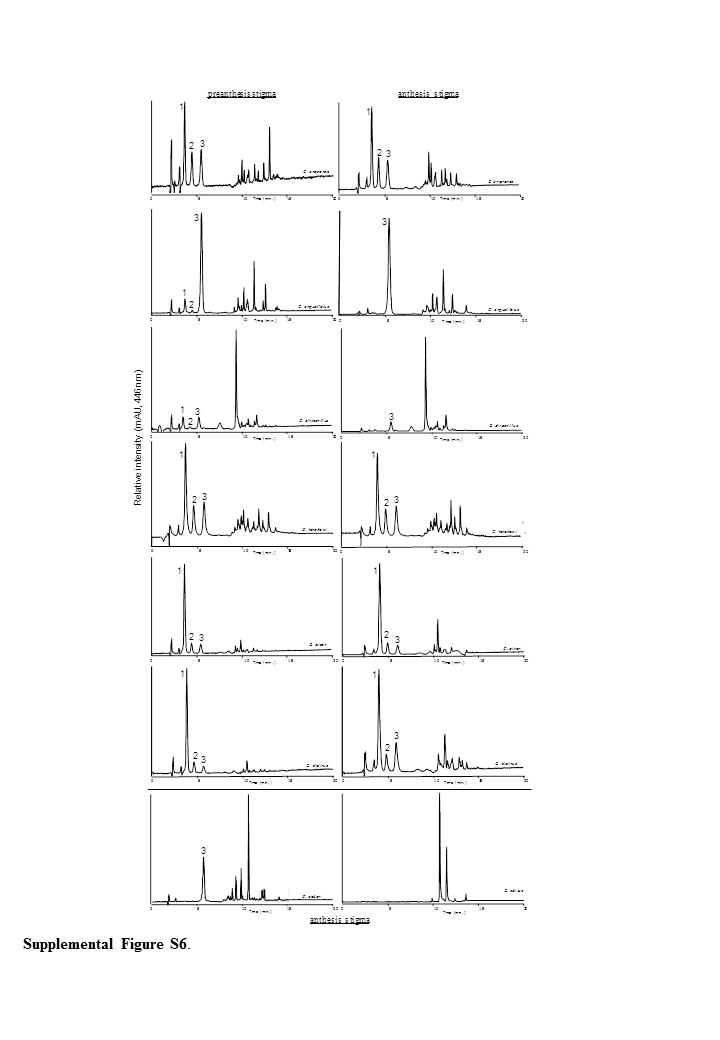

Supplement: Figure S6 — Presence of apocarotenoids in C. ancyrensis stigmas. A, In the upper part the HPLC profile of preanthesis stigma at 400 nm and below LC-PDA/UV isoplot chromatogram showing the compounds detected (200–550 nm). B, In the upper part the HPLC profile of anthesis stigma at 400 nm and below LC-PDA/UV isoplot chromatogram showing all the compounds detected (200–550 nm). C, Representative HPLC chromatograms from aqueous extracts of C. ancyrensis stigma (black line) and C. sativus stigma (grey line) at 446 nm. Labels correspond to compounds shown in Table 1. The red and grey circles denoted the compounds with absorbance between 424–446 nm and 379–399 nm, respectively. (TIF) [file pone.0071946.s006.tif]

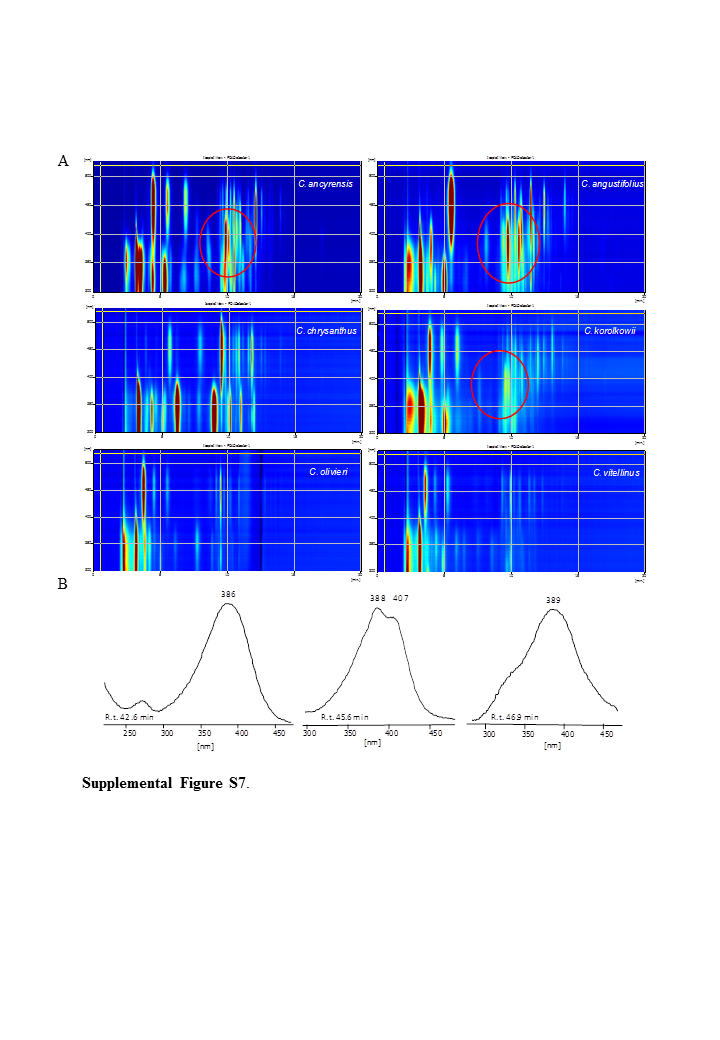

Supplement: Figure S7 — Presence of different types of apocarotenoids in the stigma extracts of several Crocus species. A. LC-PDA/UV isoplot chromatogram showing all the compounds detected (200–550 nm) of stigma aqueous extracts of different Crocus species with chromatographic method A. The red circles denoted the compounds with absorbance between 379–399. B. Absorbance spectrum of non-crocin apocarotenoids detected in the stigmas of C. ancyrensis. (TIF) [file pone.0071946.s007.tif]
