# Supplementary material for: Crocins with High Levels of Sugar Conjugation Contribute to the Yellow Colours of Early-Spring Flowering Crocus Tepals
Source: PLoS One. 2013 Sep 13;8(9):e71946. doi: 10.1371/journal.pone.0071946 (PMC3772802; doi:10.1371/journal.pone.0071946)
Supplement: Table S1 — Flowering time, tepal color and distribution of Crocus species from section Nudiscapus. The most recent classification is from Mathew (Mathew, 1982). He divided section Nudiscapus in nine series based on the division of the style, corm tunic features and flowering time. Crocus species with yellow tepals are highlight in yellow. (DOC) [file pone.0071946.s008.doc]

**Supplementary Table S1**. Flowering time, tepal color and distribution of *Crocus* species from section Nudiscapus. The most recent classification is from Mathew (1982). He divided section Nudiscapus in nine series based on the division of the style, corm tunic features and flowering time. *Crocus* species with yellow tepals are highlight in yellow.

| ***Crocus* Species**  **Section Nudiscapus** | **Flowering time** | **Tepal colour** | **Distribution** |
| --- | --- | --- | --- |
| **Series *Reticulati*** |  |  |  |
| *C. reticulatus* | spring | white or lilac | Italy; Croatia; Serbia; Macedonia; Bulgaria; Hungary; Romania; Crimea; Caucasus; Turkey |
| *C. sieberi* | spring | shades of lilac blue, yellow throat | Greece; Macedonia; Croatia; Albania; Bulgaria |
| *C. dalmaticus* | spring | shades of lilac, often with variously marked yellowish outer petals, yellow throat | Croatia; Bosnia; Albania |
| *C. robertianus* | autumn | lilac, often quite pale | Greece |
| *C. abantensis* | spring | distinctive blue colour | Turkey |
| *C. ancyrensis* | spring | yellow | Turkey |
| *C. cvijicii* | spring | yellow | Serbia; Albania; Macedonia; Greece |
| *C. gargaricus* | spring | yellow | Turkey |
| *C. angustifolius* | spring | yellow marked purple | Crimea, Ukraine, Armenia |
| *C. sieheanus* | spring | bright orange yellow | Turkey |
| *C. rujanensis* | spring | mid to deep purple inside, but with a cream to buff exterior | Albania; Macedonia |
| *C. cancellatus* | autumn | white to blue violet | Turkey; Lebanon; Israel; Iran; Irak |
| *C. hermoneus* | aurumn | pale lilac | Israel, Jordan |
| **Series *Biflori*** |  |  |  |
| *C. biflorus* | spring | deep violet/blue; Violet with broad white corona around yellow throat; pale lilac | Turkey, Greece, Sicily, Italy |
| *C. chrysanthus* | spring | yellow sometimes marked with bronze/purple | Turkey |
| *C. danfordiae* | spring | white, pale yellow or light blue-violet | Turkey |
| *C. almehensis* | spring | yellow sometimes marked with bronze/purple | Iran |
| *C. cyprius* | spring | light lilac with dark stain on petals | Cyprus |
| *C. hartmannianus* | spring | lilac or white, stained violet on outer petals | Cyprus |
| *C. aerius* | spring | pale violet eined flowers | Turkey |
| *C. pestalozzae* | spring | blue or white with a yellow throat | Turkey |
| *C. caspius* | autumn | white often stippled lilac on outer petals | Iran |
| *C. kerndorffiorum* | spring | lilac, exterior of outer petals creamy with central stripe | Turkey |
| *C. paschei* | spring | flowers shades of lilac, exterior of outer petals silvery of buff colored | Turkey |
| *C. wattiorum* | autumn | lilac | Turkey |
| *C. adanensis* | spring | pale lilac | Turkey |
| *C. leichtlinii* | spring | pale lilac often stained grey/blue near base/yellow throat | Turkey |
| **Series *Orientales*** |  |  |  |
| *C. alatavicus* | spring | white | Central Asian mountains |
| *C. korolkowii* | spring | yellow flower sometimes marked with bronze/purple | Afghanistan; Pakistan; Tajikistan; Uzbekistan |
| *C. michelsonii* | spring | whitish heavily marked with lilac blue on the outside of the petals | Turkmenistan; Iran |
| **Series *Flavi*** |  |  |  |
| *C. flavus* | spring | yellow flowers, sometimes with brownish exterior markings | Serbia; Greece; Bulgaria; Romania; Turkey |
| *C. olivieri* | spring | orange-yellow usually marked with purplish brown | Turkey |
| *C. antalyensis* | spring | lilac or white, often with exterior markings | Turkey |
| *C. candidus* | spring | white, yellow throat, outer petals usually speckled grey/purple | Turkey |
| *C. vitellinus* | spring | yellow with a distinct orange zone in the throat | Turkey; Syria |
| *C. graveolens* | spring | Yellow, variously marked with brown/purple. | Turkey; Syria; Lebanon; Israel |
| *C. hyemalis* | spring | White, slightly flecked with varying amounts of violet on the reverse. Internally it has a deep golden, to almost orange | Israel; Lebanon |
| **Series *Aleppici*** |  |  |  |
| *C. aleppicus* | winter | white creamy flowers | Syria |
| *C. veneris* | autumn | white with some purple veining on outer petals | Cyprus |
| *C. boulosii* | winter | white flowers with some dark markings at base of outer petals | Libya |
| **Series *Carpetani*** |  |  |  |
| *C. carpetanus* | spring | pale lilac or whitish flower | Spain and Portugal |
| *C. nevadensis* | spring | white, cream or pale lilac with violet veins | Africa; Spain |
| **Series *Intertexti*** |  |  |  |
| *C. fleischeri* | spring | white with purple markings at base of outer petals | Turkey; Greek islands |
| **Series *Speciosi*** |  |  |  |
| *C. speciosus* | autumn | lilac blue, veined darker | Crimea; Caucasus; Iran; Turkey |
| *C. pulchellus* | autumn | pale to mid lilac with darker veins | Macedonia; Serbia; Bulgaria; Greece; Turkey |
| **Series *Laevigatae*** |  |  |  |
| *C. laevigatus* | autumn- early spring | lilac or white usually with a dark vein or veins. | Greece |
| *C. tournefortii* | autumn | pale-dark lilac | Greece; Crete |
| *C. boryi* | autumn | large creamy white flower | Greece |

Mathew, B., 1982. The crocus - A revision of the Genus crocus, London.
